# A Potential Information Capacity Index for Link Prediction of Complex Networks Based on the Cannikin Law

**DOI:** 10.3390/e21090863

**Published:** 2019-09-04

**Authors:** Xing Li, Shuxin Liu, Hongchang Chen, Kai Wang

**Affiliations:** National Digital Switching System Engineering and Technological R&D Center, Zhengzhou 450002, China

**Keywords:** link prediction, complex networks, information capacity, Cannikin Law

## Abstract

Recently, a number of similarity-based methods have been proposed for link prediction of complex networks. Among these indices, the resource-allocation-based prediction methods perform very well considering the amount of resources in the information transmission process between nodes. However, they ignore the information channels and their information capacity in information transmission process between two endpoints. Motivated by the Cannikin Law, the definition of information capacity is proposed to quantify the information transmission capability between any two nodes. Then, based on the information capacity, a potential information capacity (PIC) index is proposed for link prediction. Empirical study on 15 datasets has shown that the PIC index we proposed can achieve a good performance, compared with eight mainstream baselines.

## 1. Introduction

Recently, more and more complex systems have been analyzed through theories of network science [1,2,3,4,5]. As an important hot topic of complex networks, link prediction [6] aims to predict the likelihood that a link exists between two nodes of complex networks. It plays an important role in recommending friends of online social networks [7] and discovering missing interactions of protein–protein interaction networks [8].

In the last few years, there are many link prediction methods for predicting missing links of complex networks. Among these methods, topology-based similarity indices are simple and effective, which attract the attention of scholars in various fields [9]. As the simplest method of similarity indices, common neighbor (CN) index measures the similarity between two endpoints by calculating the number of common neighbors between them [10]. Based on CN, many common-neighbor-based methods have been proposed through weighing the common neighbors by local information, such as Adamic–Adar (AA) index [11], resource allocation (RA) index [12], CAR [13] and so on. These local indices perform very well in many types of networks, but they need more topology information to improve the prediction accuracy in some networks. Considering longer paths, Local Path (LP) index [14] and Extended Resource Allocation (ERA) index [4] are proposed by adding the paths with length 3 to the CN index and RA index, respectively. Furthermore, many global indices are proposed by considering all the topological information between two endpoints, such as Katz index [15], SimRank [16], Average Commute Time (ACT, also called Mean Commute Time) [17,18] and Cosine Similarity Time (Cos+) [19]. In the real prediction, the global indices perform better than most of other methods, but they are not suitable for large-scale networks due to their high complexity. It is worth mentioning that, considering the coupling information of local topology, some resource-allocation-based indices and local-path-based indices perform well or even better than global indices [9]. These indices considering coupling information of local topology are very suitable for large-scale network prediction, because their complexity is higher than that of CN but lower than that of global indices. Although these indices can get a good performance with lower complexity, most of them ignore the potential information capacity between endpoints.

**Figure 1:**
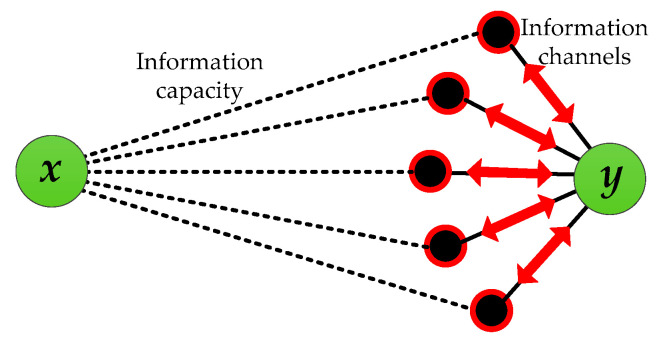
The potential information capacity between endpoints.

In the real world, various types of information are transmitted constantly in different networks [20]. Any neighbor of a node can be regarded as the anchor point of information channel for information transmission, and the information capacity denotes the information transmission capability for any information channel (as shown in Figure 1). For online social networks, the larger the potential information capacity between two users *x* and *y*, the greater the likelihood that hot topics (such as rumors, news, stocks, etc.) will spread between them [21]. That is, they are more likely to be friends. According to mainstream indices (such as RA index and LP index), the similarity between nodes is mainly focused on considering common neighbors and their related paths. However, if node *y* receives or sends information to node *x*, the number of neighbor nodes Γ(y) and the information transmission capability between *x* and Γ(y) determine the capability of information exchanged between them through Γ(y). Therefore, besides the local information considered by the existing indices, information channels (all the neighbors, not the common neighbors) and their information capacities are also play an important role in describing the similarity between two endpoints.

In view of the above analysis, a potential information capacity (PIC) index is proposed for link prediction. To quantify the information transmission capability between any two nodes, the information capacity is defined based on the Cannikin Law. With a parameter adjusting the strength of potential information capacity for different networks, the PIC index measures the similarity between two endpoints by considering the information channels and their information capacity. Experimental results show that the PIC index proposed can improve the prediction accuracy of 15 datasets, compared with several global and local indices.

The main parts of this paper are organized as follows: in Section 2, the information capacity is defined and the potential information capacity index is introduced; in Section 3, two standard metrics and eight mainstream baselines are described; in Section 4, all the 15 datasets and their topological features are introduced; in Section 5, the comparison between PIC index and eight mainstream methods is discussed; finally, a brief conclusion is given.

## 2. The Potential Information Capacity Index

The information transmission or interaction process (including resource transmission) between nodes has been described and used by several link prediction methods, and their prediction accuracy is also very high. However, they ignore the analysis and utilization of the potential information capacity between nodes. In this section, motivated by the Cannikin Law, we propose an information capacity quantification method and a new similarity index.

### 2.1. Information Capacity Based on the Cannikin Law

Information transmission is a common phenomenon in nature and human society, and it is also an important intrinsic motivation for establishing connections in complex networks [12]. Different kinds of information flow constantly in different networks, such as messages are sent from the terminal to any person through the infrastructure network [22], passengers travel from one train station to another through the railway transportation network [23], neural signal is transmitted from one neuron to another through the neural network [24] and so on.

**Figure 2:**
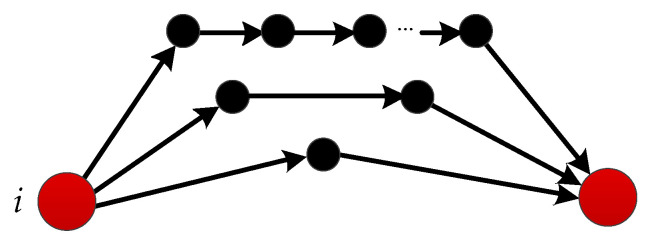
The process of information transmission between nodes through multiple paths.

As shown in Figure 2, if node *i* has one unit of information, and will transfer it to node *j* through self-avoiding random walk on any path in multipath Path*_ij_*, the amount of information $R_{ij}$ received by *j* can be expressed as:

(1) $$R_{ij}=\prod_{z\in Path_{ij}}\frac{1}{k_{z_{ij}}-1}$$

here, $k_{z_{ij}}$ denotes the node degree of vertex $z_{ij}$, where $z_{ij}$ is the node on the path (Path*_ij_*). Obviously, $R_{ij}$ represents the ability to transmit information between nodes *i* and *j*.

Considering the transmission fading and computational complexity of multi-hop paths, we just analyze the information transmission process of paths below two hops [25]. Therefore, the amount of information received by *j* through a certain common neighbor *z_ij_* can be expressed as:

(2) $$R_{ij}=\frac{1}{k_{z_{ij}}-1}$$

After estimating the amount of information transmitted through common neighbors, we are wondering how to use the process of information transmission to define or quantify the information capacity between any two nodes. In the real information transmission process, since the high-degree common neighbor is more easily selected as the transmission relay node [26], the information capacity between two endpoints is strongly related to the information transmission capability of high-degree common neighbors.

**Figure 3:**
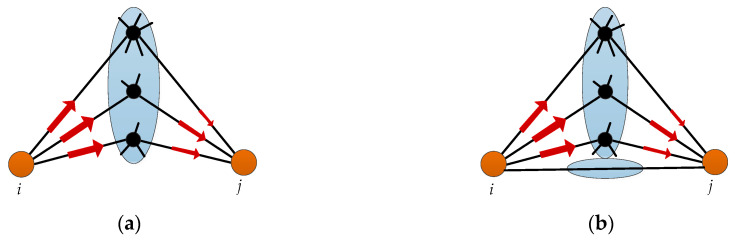
The information capacity between nodes based on the Cannikin Law. (**a**) Nodes *i* and *j* without a direct connection; (**b**) Nodes *i* and *j* with a direct connection.

**Figure 4:**
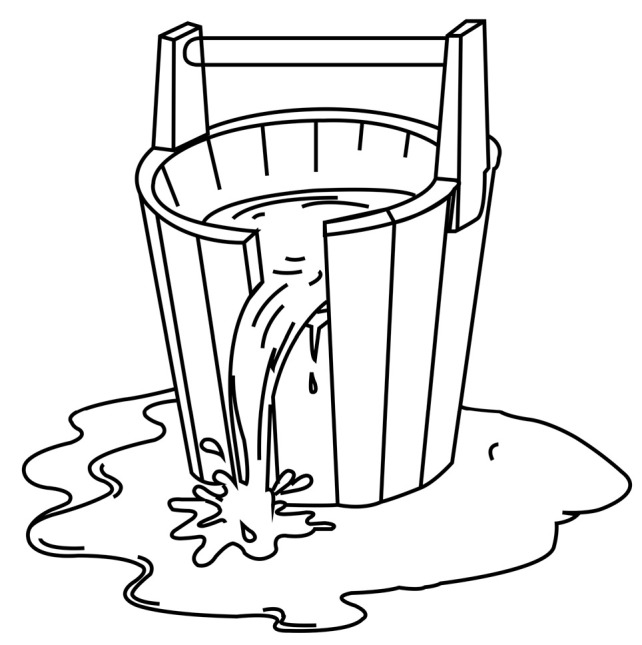
The sketch map of the Cannikin Law.

As shown in Figure 3a, if all the paths between any two nodes *i* and *j* are compared to one “container” (a bucket for storing information), the capacity of the container indicates the potential information transmission capability between nodes through various possible paths. According to the theory of the Cannikin Law [27], the capacity of the wooden bucket is limited by the height of its shortest plank (as shown in Figure 4). In different type of complex networks, information flow can be traffic flow in traffic networks, topic flow in social networks, or bioelectricity flow in neural networks. As the special kind of material flow, information flow also has the common characteristics and attributes of fluid, which can also be described and studied by the Cannikin Law. Based on the above theory, each path between nodes *i* and *j* can be regarded as a plank of a wooden bucket. Then, their information capacity IC(i,j) is determined by the number of paths *n_ij_* (number of planks) and the minimum amount of information $R_{ij}^{Min}$ transmitted by these paths (transmitted by the shortest plank), which can be expressed as:

(3) $$\begin{array}{cc} IC(i,j) & ={\left(n_{ij}\cdot R_{ij}^{Min}\right)}^{\beta }\\ & ={\left(\frac{n_{ij}}{k_{z_{ij}}^{\max}-1}\right)}^{\beta } \end{array}$$

here, $k_{z_{ij}}^{\max}$ denotes the highest node degree of common neighbors between *i* and *j*, and β≥0 aims to adjust the strength of information transmission capability for different types of networks.

If there is a direct connection between two endpoints *i* and *j*, as shown in Figure 3b, the information capacity IC(i,j) can be expressed as (the direct connection *v_ij_* can be regarded as another bucket with only one piece of plank):

(4) $$\begin{array}{cc} IC(i,j) & ={\left(1\cdot 1+n_{ij}\cdot R_{ij}^{Min}\right)}^{\beta }\\ & ={\left(1+\frac{n_{ij}}{k_{z_{ij}}^{\max}-1}\right)}^{\beta } \end{array}$$

Taking account of the two cases in Figure 3a,b, we make a definition of information capacity between any two endpoints in complex network.

**Definition** **1.** Considering a pair of endpoints *i* and *j* in complex network, $z_{ij}^{\prime }$ is the common neighbor of them. The information capacity IC(i,j) between the two endpoints, which represents the information transmission capability between them, can be quantified as:

(5) $$IC(i,j)={\left(a_{ij}+\frac{n_{ij}}{k_{z_{ij}^{\prime }}^{\max}-1}\right)}^{\beta }$$

*a_ij_* is the element value of the adjacency matrix ***A***, which denotes whether there is a connection between nodes *i* and *j*.

### 2.2. The Potential Information Capacity Index

Consider an undirected network *G*(*V*, *E*), where *V* and *E* are the sets of vertices and edges, respectively. Given a link prediction method, it assigns a score *s_xy_* to each pair of endpoints *x* and *y*. The score *s_xy_* can be a measure of the similarity between two endpoints, and the score for each nonexistent link represents the likelihood that the link exists.

**Figure 5:**
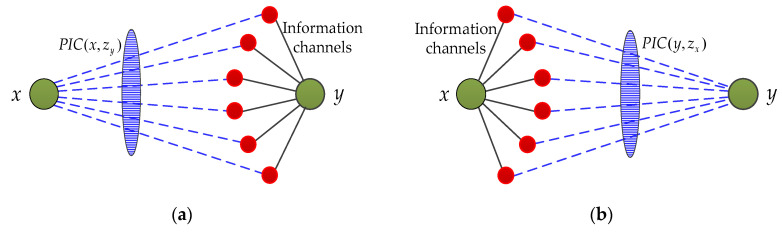
The potential information transmission between nodes through neighbors based on information capacity. (**a**) Information transmission between *x* and *z_y_*; (**b**) Information transmission between *y* and *z_x_*.

In general, the simplest way to calculate the likelihood that a link exists between two endpoints is to directly use the information capacity between them. However, it will ignore the important role of neighbor nodes in the potential information transmission process. In the real world, any node is transmitting information through its neighbor nodes. As shown in Figure 5a, the neighbor node *z_y_* can be regarded as antennas of the node *y*, which is the anchor point of information channels for receiving and transmitting information. Theoretically, the calculation of all the potential information capacity between two endpoints should consider all the information channels and their information capacity at the same time. Based on the above discussion, the similarity between two endpoints is calculated by the information capacity between their neighbors and endpoints.

**Definition** **2.** Considering a pair of nodes, x,y∈V. *z_x_* is the neighbor of node *x*, and *z_y_* is the neighbor of node *y*. Considering information channels and their information capacity, the potential information capacity (PIC) index composes of all the potential information capacity between nodes *x* and *z_y_*, nodes *y* and *z_x_*, which can be defined as:

(6) $$\begin{array}{l} s_{xy}^{PIC}=\sum_{z_{y}\in \Gamma (y)}IC(x,z_{y})+\sum_{z_{x}\in \Gamma (x)}IC(y,z_{x})\\ =\sum_{z_{y}\in \Gamma (y)}{\left(a_{xz_{y}}+\frac{n_{xz_{y}}}{k_{z_{xz_{y}}^{\prime }}^{\max}-1}\right)}^{\beta }+\sum_{z_{x}\in \Gamma (x)}{\left(a_{yz_{x}}+\frac{n_{yz_{x}}}{k_{z_{yz_{x}}^{\prime }}^{\max}-1}\right)}^{\beta } \end{array}$$

when β=0, the PIC index becomes $s_{xy}^{PIC}=k_{x}+k_{y}$, which is similar to PA index ($s_{xy}^{PA}=k_{x}\cdot k_{y}$). Obviously, the complexity of PIC index is between $O(N{\langle k\rangle }^{2})$ (PA) and $O(N{\langle k\rangle }^{3})$ (LP).

Considering that the neighbor node is the anchor of information channels for the node to exchange information, the physical meaning of the Equation (6) is that the potential information capacity between any two endpoints is the sum of the information capacity of all possible information channels. That is, the potential information capacity composes of all the information capacity between neighbor nodes of one endpoint and the other endpoint.

## 3. Metrics and Baselines

### 3.1. Metrics

Two standard metrics are widely used to quantify the accuracy of link prediction methods: area under the receiver operating characteristic curve (AUC) [28,29] and precision [30,31]. In principle, a link prediction method gives each non-observed link a similarity score to quantify its existence likelihood. The AUC evaluates the method’s performance as a whole while the precision only focuses on the *L* links with top ranks or highest scores. A detailed description of these two metrics is as follows.

Given the ranking of all non-observed links, the AUC value can be interpreted as the probability that the score given to a randomly chosen missing link is higher than a randomly chosen non-existent link [6]. In the algorithm implementation, we usually calculate the score of each non-observed link instead of giving the ordered list since the latter task is more time consuming. At each time, we randomly pick a non-existent link and a missing link to compare their scores, if among *n* times of independent comparisons, there are $n^{\prime }$ times the missing link having a higher score and $n^{\prime \prime }$ times they have the same score, the AUC value of the method is:

(7) $$\mathrm{AUC}=\frac{n^{\prime }+0.5n^{\prime \prime }}{n}$$

Obviously, if all the scores are generated from an independent and identical distribution, AUC≈0.5. An AUC score of 1.0 represents a perfect prediction while a random method has a score of 0.5. Therefore, the extent to which a link prediction method exceeds 0.5 indicates how much better its prediction accuracy than pure chance.

Precision only pays attention to the top-ranked links. In practice, all non-observed links are ranked in descending order according to their similarity scores. The precision is defined as the ratio of relevant items selected to the number of items selected [30]. That means if we take the top-*L* links as the predicted ones, among which *m* links belong to missing links, then the precision value is defined as:

(8) $$\mathrm{Precision}=\frac{m}{L}$$

Obviously, the precision value is related to the parameter *L*. For a given *L*, the higher precision value means better performance. In practice, *L* is generally set to 100 for large-scale networks, such as Ref. [4,32]. Thus, in order to compare the results more intuitively and clearly in multiple datasets, we set *L*=100 in this paper.

### 3.2. Baselines

We compare the PIC index with eight mainstream similarity indices, including five local indices: CN, AA, CAR, RA and LP index, and three global indices: Katz, ACT and Cos+ index. A brief description of these indices is shown as follows:

1. Common Neighbor (CN) index [10] calculates the similarity of two endpoints by the number of their common neighbors:
  (9) $$s_{{}_{xy}}^{CN}=|\Gamma (x)\cap \Gamma (y)|$$
  Γ(x) is the set of neighbors of node *x*, and Γ(x)∩Γ(y) represents the common neighbors between nodes *x* and *y*.
2. Resource Allocation (RA) index [12] measures the similarity of two endpoints by the received resource (information) of endpoint *y* through common neighbors sending by endpoint *x*:
  (10) $$s_{{}_{xy}}^{RA}=\sum_{z\in |\Gamma (x)\cap \Gamma (y)|}\frac{1}{k_{z}}$$
  $k_{z}$ denotes the node degree of common neighbor *z*.
3. Adamic–Adar (AA) index [11] weights the common neighbors according to the node degree, and punishes the common neighbors with big degree:
  (11) $$s_{{}_{xy}}^{AA}=\sum_{z\in |\Gamma (x)\cap \Gamma (y)|}\frac{1}{\log k_{z}}$$
  This method considers that the contribution of common neighbors with low node degree are weighted higher than that of nodes with high node degree, and the weighting scheme used by AA index is the reciprocal of the logarithm of node degree [10].
4. CAR index [13] believes that the link is more likely to exist between two nodes if their common-first-neighbors are members of a strongly inner-linked cohort:
  (12) $$s_{{}_{xy}}^{CAR}=|\Gamma (x)\cap \Gamma (y)|\cdot \sum_{z\in |\Gamma (x)\cap \Gamma (y)|}\frac{\left|\gamma (z)\right|}{2}$$
  γ(z) denotes the sub-set of the neighbors of node *z*, and all these neighbors of node *z* are also the common neighbors of nodes *x* and *y*.
5. Local Path (LP) index [14] considers the longer paths with length 3 between endpoints based on the common neighbors:
  (13) $$S=A^{2}+\alpha \cdot A^{3}$$
  α denotes the adjust parameter for longer paths, and ***A*** is the adjacency matrix.
6. Katz index [15] calculates the similarity between two nodes by considering all the paths between them:
  (14) $$s_{{}_{xy}}^{Katz}=\sum_{l=1}^{\infty }\varepsilon^{l}\cdot |path_{xy}^{l}|=\varepsilon A_{xy}+\varepsilon^{2}{\left(A^{2}\right)}_{xy}+\varepsilon^{3}{\left(A^{3}\right)}_{xy}+\ldots$$
  here, ε is the adjust parameter for paths, and $path_{xy}^{l}$ is the set of paths with length *l* between nodes *x* and *y*.
7. Average Commute Time (ACT) [17] calculates the similarity between two nodes by the average number of steps required by random walks between them:
  (15) $$s_{{}_{xy}}^{ACT}=\frac{1}{l_{xx}^{+}+l_{yy}^{+}-2l_{xy}^{+}},$$
  $L^{+}$ denotes the pseudo-inverse of matrix ***L*** = ***D*** − ***A***, and $l_{xy}^{+}$ is the corresponding entry in $L^{+}$.
8. Cosine Similarity Time (Cos+) [19] calculates the similarity between nodes based on the angle between the random walk vectors:
  (16) $$s_{{}_{xy}}^{Cos+}=\frac{v_{x}^{T}v_{y}}{\left|v_{x}|\cdot |v_{y}\right|}=\frac{l_{xy}^{+}}{\sqrt{l_{xx}^{+}\cdot l_{yy}^{+}}}.$$

## 4. Data

To test the effectiveness of the proposed PIC index, twelve different real networks and three synthetic dynamic networks (randomly generated by BA model with different scales, denoted as SD-1, SD-2, SD-3 respectively) are used in our experiments. The twelve real networks are introduced as follows: (i) AIDS-Blog (AIDS) [33]: a citation network among blogs related to AIDS, patients, and their support networks. (ii) Food Web of Florida Bay ecosystem (FWFB) [34]: the network of carbon exchanges occurring during the wet season in Florida Bay. (iii) Food Web of Everglades ecosystem FWEW [35]: the network of carbon exchanges occurring during the wet season in the cypress wetlands of South Florida. (iv) Caenorhabditis elegans (CE) [36]: the neural network of the nematode worm. (v) Email [37]: the internal email communication network between employees of a mid-sized manufacturing company. (vi) Political blogs (PB) [38]: a political blog network of USA. (vii) Hamster [39]: a friendship network between users on the website hamsterster.com. (viii) Figeys [40]: a protein–protein interaction network of Humans (*Homo sapiens*). (ix) UcSocial [41]: a communication network between students in online community from the University of California, Irvine through messages. (x) OpenFlights (Flight) [42]: the flight network between global airports. (xi) Yeast PPI (Yeast) [43]: a network of interactions between proteins of yeast. (xii) Haggle [44]: a network of contacts between people measured by carried wireless devices.

The basic topological features of the 15 datasets are shown in Table 1. Each original data is randomly divided into training set contains 90% of links, and the probe set contains the remaining 10%.

## 5. Results

### 5.1. AUC Results

**Figure 6:**
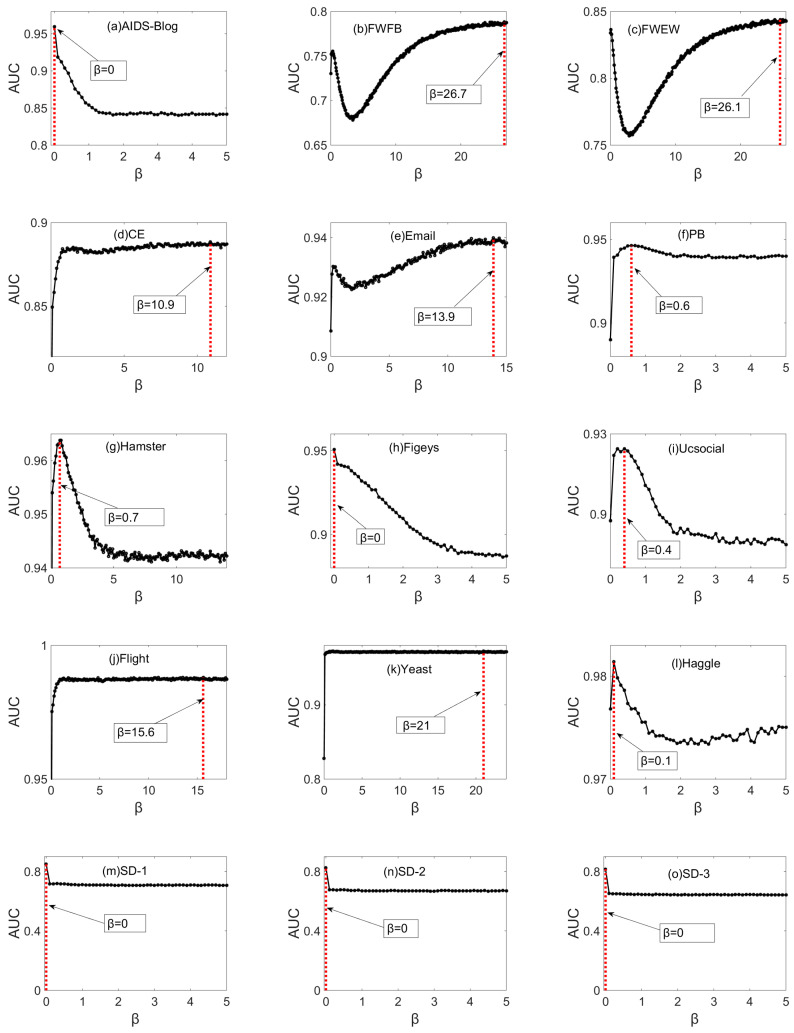
The AUC values of PIC index on 15 datasets with different values of β. Each AUC value is the average of 20 realizations, each of which corresponds to an independent division of $E^{T}$ and $E^{P}$.

Firstly, let us explore the AUC results of the PIC index with different β in 15 datasets, and each result is the average of 20 realizations. With the changing of parameter β, the AUC values are continuous varies for 15 datasets as shown in Figure 6. For most of the datasets, the values of the PIC index are very high when the parameter 0≤β<1 (except FWFB, FWEW and Email). Similarly, when the adjust parameter β is equal to or very close to zero, the AUC value of some datasets can obtain the maximum value, which indicates that the link establishment of these networks considers more about information channels. However, for some datasets such as FWFB, FWEW, CE, Email, Flight and Yeast, the AUC value get the maximum value when the parameter β is far greater than 1, which indicates that the link establishment of these networks considers more about information capacity of information channels.

Table 2 shows the comparison of the AUC value between PIC index and eight mainstream similarity indices. PIC-Max is the maximum AUC value of PIC index, and PIC-0.9 denotes the AUC value with parameter β=0.9. In 14 out of 15 networks, the AUC value of PIC index is the highest, and only lower than the Cos+ in the Flight network. Having only considered the number of common neighbors between endpoints, CN gets the lowest AUC value for most of networks. The performance of common-neighbor-based indices such as AA and CAR is better than CN, even better than global indices in some networks. For all the networks except SD-3, the AUC value of RA is generally higher than that of CN. Obviously, the resource transmission process describing the common neighbor based on resource allocation can achieve better prediction results than directly calculating the number of common neighbors (CN). It also indicates that the contribution of different common neighbors to similarity is different for most complex networks.

With the longer paths considered, LP index obtains a good performance by adding a little more complexity. Obviously, the global indices can achieve a better performance than local indices especially Katz with the highest complexity. However, the AUC values of ACT and Cos+ are lower than expected in the three synthetic dynamic networks, probably because these indices are not suitable for datasets with power-law distribution. Interestingly, for Yeast, the AUC value of Katz, Cos+ and PIC are the same. This phenomenon shows that the path information above the third-order in the current network has little effect on the probability that a link exists between nodes, and the AUC values of these indices are very similar due to the similarity of the different coupling calculation of these special local topological structures (because the average path of the network is long, but the clustering coefficient is high). In addition, the parameter of these indices has little effect on the result, and when the parameter value is small, it has achieved a higher value and remains stable (as shown in Yeast in Figure 6).

As can be seen, having considered all the information channels and their information capacity, the PIC index can perform even better than these mainstream baselines in real networks or synthetic dynamic networks. In many networks such as AIDS, FWFB, FWEW, Hamster, Figeys, UcSocial, SD-1, SD-2 and SD-3, the PIC index is significantly higher than other methods. Compared with these local indices, the performance of the PIC index is increased by 2% to 68% under the AUC metric, while compared with global indices, the performance is improved by up to about 2.18 times (the AUC value of Cos+ in SD is very small). Overall, the average improvement rate of the PIC index is about 12.25% compared to these baselines, with a maximum improvement rate of 68%. Furthermore, the higher AUC results of PIC index show that the potential capacity of information transmission among nodes can represent the similarity between nodes to some extent. In addition, the parameter *β* is recommended to be set around 0.9 for PIC index under the AUC metric in the real prediction, and most of these AUC values are still higher than other indices (see PIC-0.9 of Table 2).

### 5.2. Precision Results

**Figure 7:**
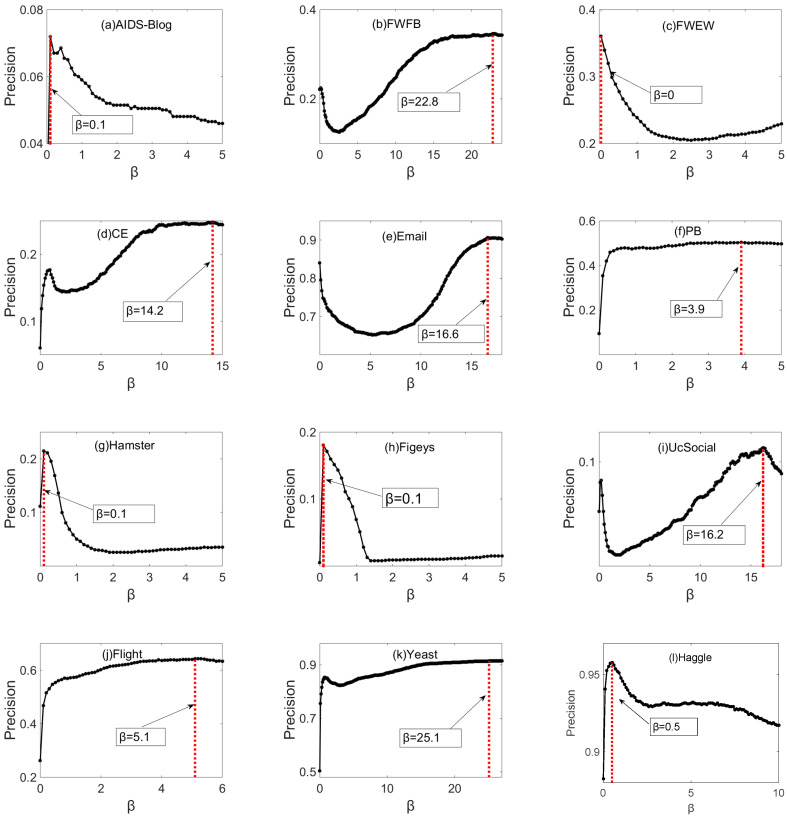

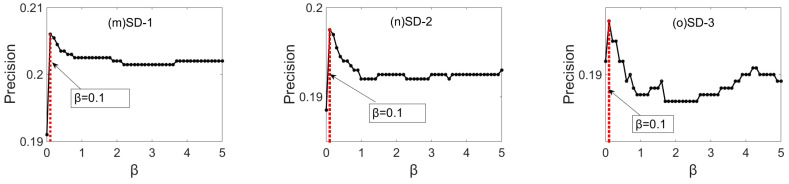
The precision values of PIC index on 15 datasets with different values of β. Each precision value is the average of 20 realizations, each of which corresponds to an independent division of $E^{T}$ and $E^{P}$.

To test the effectiveness of the PIC index more deeply, the standard metric precision is introduced to measure the prediction accuracy from another perspective. As shown in Figure 7, there are 15 precision results with the change of *β* for different datasets. Same as the AUC results, the precision values of the PIC index are also very high when 0 ≤ *β <* 1 for most networks (except FWFB, CE, Email and UcSocial). The precision value of some datasets can achieve the maximum value when the parameter β is around 0, which indicates that the establishment of top *L* predicted links in these networks considers more about information channels. However, for other datasets such as FWFB, CE, Email, PB, UcSocial, Flight, and Yeast, the precision value gets the maximum value when the parameter β is far greater than 1, which indicates that the establishment of top *L* predicted links in these networks considers more about the information capacity of information channels.

Table 3 shows the comparison of precision between PIC index and eight mainstream baselines. PIC-Max is the maximum precision value of PIC index, and PIC-0.4 denotes the precision value with parameter β=0.4. For all the 15 datasets, PIC-Max can obtain the best performance under the standard metric precision. Similarly, in 14 out of 15 networks, PIC-0.4 gets the best performance, and only worse than CAR, LP and Katz in Flight network. Surprisingly, the precision value of CN index is higher than RA and AA for many datasets such as FWFB, PB, UcSocial, Flight, Haggle, SD-1, SD-2, and SD-3. For most of datasets, LP and Katz achieve a better performance than these common-neighbor-based local indices with longer paths considered. However, the precision values of ACT and Cos+ are lower than all the indices, probably because they are not suitable for the standard metric precision. For all the local and global indices, the precision results in most networks (except AIDS, PB, UcSocial, Haggle and SD-1) are significantly improved by the proposed PIC index, and the precision value is increased by 0.11 to 0.91. Overall, PIC index can increase the precision value by an average of 0.19 and by a maximum of 0.95 compared to these baselines (because the precision values of ACT and Cos + were close to 0). The proposed PIC index performs very well in all the datasets under precision metrics, which indicates that the potential information capacity between two endpoints is positively related to the establishment of top *L* predicted links. In addition, under the standard metric precision, we recommend that the parameter β is set at around 0.4 in the real prediction for most of the datasets.

## 6. Conclusions

Topology-based similarity indices play an important role in predicting missing links of large-scale networks. Motivated by the potential information capacity between two endpoints, a potential information capacity index is proposed for link prediction. Based on the Cannikin Law, the information capacity considers the number of paths (number of planks) and the minimum amount of information transmitted by these paths (shortest plank). The PIC index can achieve a good performance with an adjust parameter of information capacity for each channel. It can obtain the maximum value for different networks under different parameter values. For most datasets, the AUC and precision of the PIC index are very close to the maximum when the parameter β is around 0.9 and 0.4. According to the PIC index, when the parameter is equal to zero, it is similar to PA index. This indicates that if the parameter is closer to zero when obtaining the maximum value for the dataset, the degree distribution of this dataset is closer to power-law. Due to its good performance in different types of real networks and low time complexity, the PIC index can be applied to many real networks, especially large-scale networks. In our future work, we will address how to quantify the information capacity between nodes in directed networks, and then propose an effective link prediction method for directed networks. Furthermore, for information networks, technology networks and other related networks, transmission nodes are subject to attack and failure in the process of real information transmission [45,46], and transmission delay varies with topology, which is also a common phenomenon [47]. Therefore, considering the above factors in the transmission process, re-modeling the information capacity between nodes will provide a new idea for link prediction.

## Figures and Tables

**Table 1:** The basic topological features of 15 datasets including 12 real networks and three synthetic dynamic networks (SD). |V| denotes the number of nodes, |E| is the number of edges, 〈k〉 is the average node degree. 〈d〉 indicates the average distance. *C* is the clustering coefficient. *r* is the assortativity coefficient. *H* represents the degree heterogeneity.

Datasets	|V|	|E|	〈k〉	〈d〉	*C*	*r*	*H*
AIDS	146	180	2.47	3.42	0.052	−0.725	5.99
FWFB	128	2075	32.42	1.78	0.335	−0.112	1.24
FWEW	69	880	25.51	1.64	0.552	−0.298	1.27
CE	297	2148	14.46	2.46	0.308	−0.163	1.80
Email	167	5784	69.26	1.87	0.541	−0.295	1.66
PB	1222	16717	27.36	2.74	0.361	−0.221	2.97
Hamster	1858	12534	13.49	3.39	0.090	−0.085	3.36
Figeys	2239	6432	5.76	3.98	0.040	−0.331	9.75
UcSocial	1899	13838	14.57	3.06	0.109	−0.188	3.82
Flight	2939	30501	20.75	4.18	0.255	0.051	5.22
Yeast	2375	11693	9.85	5.10	0.378	0.469	3.48
Haggle	274	2124	15.5	2.42	0.566	−0.474	3.66
SD-1	800	1727	4.32	3.14	0.211	−0.242	6.14
SD-2	1200	2527	4.21	3.27	0.172	−0.229	6.99
SD-3	2000	4123	4.12	3.40	0.144	−0.220	8.43

**Table 2:** Comparison of the AUC values between the PIC index and eight similarity indices. Each AUC value is the average of 20 realizations, each of which corresponds to an independent division of $E^{T}$ and $E^{P}$.

Datasets	CN	RA	AA	CAR	LP ^1^	LP ^2^	Katz ^1^	Katz ^2^	ACT	Cos+	PIC-0.9	PIC-Max
AIDS	0.599	0.611	0.612	0.599	0.834	0.834	0.851	0.850	0.957	0.591	**0.857**	**0.960**
FWFB	0.604	0.613	0.605	0.621	0.622	0.670	0.622	0.681	0.725	0.655	**0.733**	**0.788**
FWEW	0.693	0.709	0.700	0.693	0.713	0.736	0.712	0.743	0.787	0.505	**0.793**	**0.844**
CE	0.852	0.873	0.868	0.851	0.870	0.870	0.869	0.868	0.748	0.860	**0.883**	**0.888**
Email	0.923	0.928	0.924	0.921	0.923	0.923	0.922	0.920	0.902	0.910	**0.926**	**0.940**
PB	0.925	0.930	0.928	0.924	0.936	0.940	0.937	0.934	0.892	0.928	**0.946**	**0.946**
Hamster	0.813	0.818	0.817	0.814	0.934	0.940	0.934	0.938	0.869	0.960	**0.963**	**0.964**
Figeys	0.566	0.569	0.569	0.566	0.887	0.901	0.884	0.898	0.915	0.837	**0.931**	**0.951**
UcSocial	0.782	0.787	0.786	0.783	0.891	0.902	0.892	0.902	0.896	0.869	**0.915**	**0.924**
Flight	0.969	0.972	0.971	0.968	0.984	0.983	0.982	0.980	0.907	0.989	**0.987**	**0.988**
Yeast	0.916	0.917	0.916	0.915	0.970	0.970	0.972	0.972	0.899	0.972	**0.972**	**0.972**
Haggle	0.962	0.963	0.962	0.962	0.970	0.970	0.970	0.970	0.959	0.909	**0.976**	**0.981**
SD-1	0.646	0.647	0.649	0.647	0.708	0.709	0.705	0.704	0.571	0.267	**0.710**	**0.848**
SD-2	0.621	0.622	0.622	0.621	0.672	0.671	0.667	0.668	0.538	0.266	**0.673**	**0.825**
SD-3	0.603	0.602	0.602	0.602	0.648	0.648	0.645	0.643	0.519	0.281	**0.647**	**0.815**

^1^ In these methods, the adjust parameter α=0.001. ^2^ The adjust parameter α=0.01.

**Table 3:** Comparison of the precision values between PIC index and eight similarity indices. Each precision value is the average of 20 realizations.

Datasets	CN	RA	AA	CAR	LP ^1^	LP ^2^	Katz ^1^	Katz ^2^	ACT	Cos+	PIC-0.4	PIC-Max
AIDS	0.013	0.029	0.028	0.013	0.054	0.054	0.055	0.055	0.000	0.000	**0.069**	**0.072**
FWFB	0.085	0.081	0.083	0.084	0.092	0.124	0.092	0.129	0.000	0.032	**0.204**	**0.347**
FWEW	0.149	0.169	0.157	0.146	0.162	0.189	0.162	0.194	0.134	0.000	**0.289**	**0.361**
CE	0.133	0.127	0.138	0.138	0.140	0.141	0.140	0.140	0.000	0.074	**0.164**	**0.248**
Email	0.708	0.709	0.717	0.703	0.713	0.708	0.713	0.697	0.000	0.617	**0.746**	**0.906**
PB	0.419	0.250	0.379	0.488	0.428	0.459	0.428	0.456	0.000	0.339	**0.467**	**0.504**
Hamster	0.018	0.006	0.012	0.037	0.021	0.064	0.021	0.081	0.000	0.023	**0.169**	**0.215**
Figeys	0.008	0.008	0.008	0.024	0.008	0.009	0.008	0.008	0.000	0.007	**0.152**	**0.181**
UcSocial	0.034	0.028	0.032	0.061	0.034	0.046	0.034	0.050	0.000	0.007	**0.067**	**0.110**
Flight	0.515	0.356	0.451	0.621	0.522	0.561	0.522	0.552	0.000	0.037	**0.547**	**0.644**
Yeast	0.694	0.499	0.709	0.683	0.700	0.755	0.700	0.741	0.000	0.249	**0.836**	**0.915**
Haggle	0.892	0.890	0.889	0.882	0.894	0.933	0.894	0.944	0.000	0.823	**0.957**	**0.958**
SD-1	0.201	0.091	0.173	0.202	0.203	0.203	0.203	0.203	0.000	0.001	**0.204**	**0.206**
SD-2	0.191	0.116	0.166	0.191	0.193	0.193	0.194	0.194	0.000	0.000	**0.195**	**0.198**
SD-3	0.188	0.123	0.158	0.187	0.189	0.189	0.189	0.189	0.000	0.000	**0.191**	**0.194**

^1^ In these methods, the adjust parameter α=0.001. ^2^ The adjust parameter α=0.01.

## References

[1] Gosak M., Markovič R., Dolenšek J., Rupnik M.S., Marhl M., Stožer A., Perc M. (2018). Network science of biological systems at different scales: A review. Phys. Life Rev..

[2] Wang M., Zhao L., Du R., Wang C., Chen L., Tian L., Stanley H.E. (2018). A novel hybrid method of forecasting crude oil prices using complex network science and artificial intelligence algorithms. Appl. Energy.

[3] Interdonato R., Atzmueller M., Gaito S., Kanawati R., Largeron C., Sala A. (2019). Feature-rich networks: Going beyond complex network topologies. Appl. Netw. Sci..

[4] Liu S., Ji X., Liu C., Bai Y. (2017). Extended resource allocation index for link prediction of complex network. Phys. A Stat. Mech. Appl..

[5] Liu S., Ji X., Liu C., Guo H. (2014). A complex network evolution model for network growth promoted by information transmission. Acta Phys. Sin..

[6] Lü L., Zhou T. (2011). Link prediction in complex networks: A survey. Phys. A Stat. Mech. Appl..

[7] Lü L., Medo M., Yeung C., Zhang Y., Zhang Z., Zhou T. (2012). Recommender systems. Phys. Rep..

[8] Yerneni S., Khan K.I., Wei Q. (2018). IAS: Interaction specific GO term associations for predicting protein–protein interaction networks. IEEE ACM Trans. Comput. Biol. Bioinform..

[9] Wang P., Xu B., Wu Y., Zhou X. (2015). Link prediction in social networks: The state-of-the-art. Sci. China Inf. Sci..

[10] Mitzenmacher M. (2004). A brief history of generative models for power law and lognormal distributions. Internet Math..

[11] Adamic L.A., Adar E. (2003). Friends and neighbors on the Web. Soc. Netw..

[12] Zhou T., Lü L., Zhang Y.C. (2009). Predicting missing links via local information. Eur. Phys. J..

[13] Cannistraci C.V., Alanis-Lobato G., Ravasi T. (2013). From link-prediction in brain connectomes and protein interactomes to the local-community-paradigm in complex networks. Sci. Rep..

[14] Lü L., Jin C.H., Zhou T. (2009). Similarity index based on local paths for link prediction of complex networks. Phys. Rev..

[15] Katz L. (1953). A new status index derived from sociometric analysis. Psychometrika.

[16] Jeh G., Widom J. SimRank: A measure of structural-context similarity. Proceedings of the ACM SIGKDD International Conference on Knowledge Discovery and Data Mining.

[17] Klein D.J., Randic M. (1993). Resistance distance. J. Math. Chem..

[18] Shang Y. (2012). Mean commute time for random walks on hierarchical scale-free networks. Internet Math..

[19] Fouss F., Pirotte A., Renders J.M., Saerens M. (2007). Random-walk computation of similarities between nodes of a graph with application to collaborative recommendation. IEEE Trans. Knowl. Data Eng..

[20] Liu L., Qu B., Chen B., Hanjalic A., Wang H. (2018). Modelling of information diffusion on social networks with applications to WeChat. Phys. A Stat. Mech. Appl..

[21] Yang D., Liao X., Shen H., Cheng X., Chen G. (2018). Dynamic node immunization for restraint of harmful information diffusion in social networks. Phys. A Stat. Mech. Appl..

[22] Dzaferagic M., Kaminski N., McBride N., Macaluso I., Marchetti N. (2018). A functional complexity framework for the analysis of telecommunication networks. J. Complex Netw..

[23] Sun Q., Guo X., Jiang W., Ding H., Li T., Xu X. (2019). Exploring the node importance and its influencing factors in the railway freight transportation network in china. J. Adv. Transp..

[24] Avena-Koenigsberger A., Misic B., Sporns O. (2018). Communication dynamics in complex brain networks. Nat. Rev. Neurosci..

[25] Yao Y., Zhang R., Yang F., Tang J., Yuan Y., Hu R. (2018). Link prediction in complex networks based on the interactions among paths. Phys. A Stat. Mech. Appl..

[26] Zeng S. (2016). Link prediction based on local information considering preferential attachment. Phys. A Stat. Mech. Appl..

[27] Liu Z., Zhang H. (2009). Interpretation of cannikin law in human resource management. J. Nanning Teach. Coll..

[28] Hanely J., McNeil B. (1982). The meaning and use of the area under a receiver operating characteristic (ROC) curve. Radiology.

[29] Wu Y., Yu H., Zhang J., Liu S., Huang R., Li P. (2018). USI-AUC: An evaluation criterion of community detection based on a novel link-prediction method. Intell. Data Anal..

[30] Herlocker J., Konstann J., Terveen K., Riedl J. (2004). Evaluating collaborative filtering recommender systems. ACM Trans. Inf. Syst..

[31] Chuan P.M., Ali M., Khang T.D., Dey N. (2018). Link prediction in co-authorship networks based on hybrid content similarity metric. Appl. Intell..

[32] Wu Z., Lin Y., Zhao Y., Yan H. (2018). Improving local clustering based top-L link prediction methods via asymmetric link clustering information. Phys. A Stat. Mech. Appl..

[33] Gopal S. (2007). The evolving social geography of blogs. Societies and Cities in the Age of Instant Access.

[34] Gerould S., Higer A. (1999). U.S. geological survey program on the south Florida ecosystem. U.S. Geol. Surv. Publicaion.

[35] Ulanowicz R.E., DeAngelis D.L. (2005). Network analysis of trophic dynamics in south Florida ecosystems. US Geol. Surv. Program South Fla. Ecosyst..

[36] Watts D.J., Strogatz S.H. (1998). Collective dynamics of “small-world” networks. Nature.

[37] Guimera R., Danon L., Diaz-Guilera A., Giralt F., Arenas A. (2003). Self-similar community structure in a network of human interactions. Phys. Rev..

[38] Adamic L.A., Glance N. The political blogosphere and the 2004 US election: Divided they blog. Proceedings of the 3rd International Workshop on Link Discovery.

[39] Lü L., Pan L., Zhou T., Zhang Y.C., Stanley H.E. (2015). Toward link predictability of complex networks. Proc. Natl. Acad. Sci. USA.

[40] Ewing R.M., Chu P., Elisma F., Li H., Taylor P., Climie S. (2007). Large-scale mapping of human protein–protein interactions by mass spectrometry. Mol. Syst. Biol..

[41] Opsahl T., Panzarasa P. (2009). Clustering in weighted networks. Soc. Netw..

[42] Opsahl T., Agneessens F., Skvoretz J. (2010). Node centrality in weighted networks: Generalizing degree and shortest paths. Soc. Netw..

[43] Bu D., Zhao Y., Cai L., Xue H., Zhu X., Lu H., Zhang J., Sun S., Ling L., Zhang N. (2003). Topological structure analysis of the protein–protein interaction network in budding yeast. Nucleic Acids Res..

[44] Chaintreau A., Hui P., Crowcroft J., Diot C., Gass R., Scott J. (2007). Impact of human mobility on opportunistic forwarding algorithms. IEEE Trans. Mob. Comput..

[45] Shang Y. (2017). Consensus in averager-copier-voter networks of moving dynamical agents. Chaos Interdiscip. J. Nonlinear Sci..

[46] Shang Y. (2018). Resilient multiscale coordination control against adversarial nodes. Energies.

[47] Shang Y. (2017). On the delayed scaled consensus problems. Appl. Sci..

